# Lung injury and the cinnamon challenge: college students should beware this Internet dare

**DOI:** 10.5249/jivr.v7i1.541

**Published:** 2015-01

**Authors:** Anand N. Bosmia, Kevin J. Leon

**Affiliations:** ^*a*^Children’s of Alabama , Pediatric Neurosurgery ,Birmingham , Alabama,US.; ^*b*^Division of Pulmonology, Allergy, and Critical Care, Department of Medicine, University of Alabama at Birmingham, Birmingham, Alabama,US.

A recent article in Pediatrics warns against inhaling cinnamon powder and cites experiments that demonstrated chronic inflammation and fibrotic changes to the pulmonary tissue of rats forced to inhale cinnamon powder, and was written in response to the growing popularity of the cinnamon challenge.^1^ The cinnamon challenge has existed for nearly a decade.^2^ This dare consists of consuming one tablespoon of cinnamon powder within one minute without drinking any fluids.^3^ The cinnamon challenge has become more popular among teenagers and young adults over the past four years.^2^

According to the American Association of Poison Control Centers (AAPCC), the number of calls to poison control centers concerning teenagers ages 13 to 19 who had undertaken the cinnamon challenge increased from 51 in 2011 to 222 in 2012. ^4^ In 2013, from January 1 to July 31, 37 such exposures were reported to poison control centers. ^4^ Not all persons who intentionally abuse or misuse cinnamon powder will need medical attention. Of the 122 calls classified as “intentional misuse or abuse” during the first three months of 2012, 30 required medical attention. ^5^ However, episodes in which medical attention is warranted may be underreported.

Cinnamon is a caustic powder composed of cellulose fibers, which neither dissolve nor biodegrade in the lungs.^1^ The powder quickly dries out the mouth, which makes swallowing the powder very difficult. Coughing and burning sensations in the mouth, nose, and throat ensue. More serious symptoms include vomiting, epistaxis, and chest tightness^1^ and even atelectasis.^6^ Furthermore, the AAPCC warns that teenager with asthma or other pre-existing respiratory problems are at greater risk for developing respiratory distress from the cinnamon challenge.^5^

General internists and family practitioners who provide on-campus outpatient services to college students might encounter students with respiratory problems secondary to participating in the cinnamon challenge. Peer pressure is an appreciable factor among both children and young adults concerning participation in the cinnamon challenge.^1^ College students might undertake the challenge as part of their initiation into a student-led organization. Persons with an allergy to cinnamon or a pre-existing respiratory condition are encouraged to disclose their condition to friends or acquaintances who participate in the cinnamon challenge so that they are less likely to be pressured into following suit.

In conclusion, college students are a population at risk for respiratory complications secondary to cinnamon toxicity. Educational efforts that highlight the dangers of the cinnamon challenge should be implemented on college campuses in addition to middle school, junior high school, and high school campuses. This Internet dare is not a concern just for pediatricians, but also for general internists, family practitioners, and pulmonologists.
